# Vaginal agenesis: Experience with sigmoid colon neovaginoplasty

**DOI:** 10.4103/0971-9261.69136

**Published:** 2010

**Authors:** Jiledar Rawat, Intezar Ahmed, Anand Pandey, Tanvir R. Khan, Sarita Singh, Ashish Wakhlu, Shiv N. Kureel

**Affiliations:** Department of Pediatric Surgery, CSM Medical University, Lucknow – 226 003, India; 1 Department of Anesthesiology, CSM Medical University, Lucknow – 226 003, India

**Keywords:** Mayer–Rokitansky–Kuster–Hauser syndrome, vaginal agenesis, vaginal reconstruction

## Abstract

**Aim:**

Objective of this study is to report our experience with sigmoid vaginoplasty in adolescents.

**Materials and Methods:**

A retrospective study of children with vaginal atresia and Mayer–Rokitansky–Kuster–Hauser syndrome. The sigmoid segment was used for vaginoplasty in all the cases.

**Results:**

Eight children were studied over a period of 7 years. The postoperative complications were ileus in 2, mucosal prolapse of the neovagina in 1, and minor wound infection in 1 patient. Seven patients are on regular follow-up. All the neovaginas were patent and functional. One patient had unacceptable perineal appearance, that is, badly scarred perineum as a late complication. None of the patients had vaginal stenosis or excessive mucus discharge, during follow-up visits. Out of the 7 patients, 2 patients are sexually active and satisfied.

**Conclusions:**

Sigmoid vaginoplasty is a safe and acceptable procedure for vaginal agenesis with good cosmetic results and acceptable complications rate. Sigmoid colon vaginoplasty is the treatment of choice because of its large lumen, thick walls resistant to trauma, adequate secretion allowing lubrication, not necessitating prolonged dilatation, and short recovery time.

## INTRODUCTION

Vaginal agenesis is a congenital problem, usually seen in cases of Mayer–Rokitansky–Kuster–Hauser (MRKH) syndrome (a Müllerian duct defect, associated with vaginal agenesis and occurs in 1/4000 to 10,000 births).[1] These patients often present in adolescence with the complaint of amenorrhea. About 15% of the patients presenting to the gynecologic outpatient department with a complaint of amenorrhea have MRKH syndrome.[2] MRKH syndrome is one of the most common indications for neovaginoplasty. The other indications for a neovagina reconstruction are cloaca, intersex disorders, and the acquired cases of vaginal loss as a result of pelvic exenteration for gynecologic cancer or postpartum necrosis. Many operative and nonoperative methods of vaginal construction have been described to date. A nonoperative method (Frank technique) used for a rudimentary vagina is serial dilatation.[3] Pedunculated skin grafts with dilatation,[3] full-thickness and partial-thickness skin grafts (Abbe–McIndoe method),[3] peritoneum,[3] bladder mucosa,[3] amnion,[4] and synthetic material[5] have been used to construct the neovagina. These modalities require long-term dilatation and stenting to prevent canal closure. The use of isolated bowel segments has been shown to provide excellent results, circumventing the need for regular dilatation and providing natural lubrication.[6] Objective of this study is to describe our experience with sigmoid vaginoplasty. We present our results of sigmoid vaginoplasty performed in patients with MRKH syndrome, with emphasis on the sexual and social outcomes of these patients in a developing country scenario.

## MATERIAL AND METHODS

This was a retrospective study from 2002 to 2008. All the 8 patients included in the study were having MRKH syndrome. All the patients underwent sigmoid colon vaginoplasty under general anesthesia. Preoperative workup included physical examination, karyotyping, abdominal and pelvic ultrasonography, and endocrinological and psychologic assessments. Informed consent, after an explanation of the potential benefits and risks of sigmoid vaginoplasty and the surgical or nonsurgical alternatives to create a neovagina, was obtained from all the patients and parents. Six weeks after the procedure, patients were allowed to start intercourse as soon as they wished. No routine vaginal dilatation was advised. The patients were followed for the effectiveness of surgery and complications, if any.

### Surgical technique

**Figure 1:**
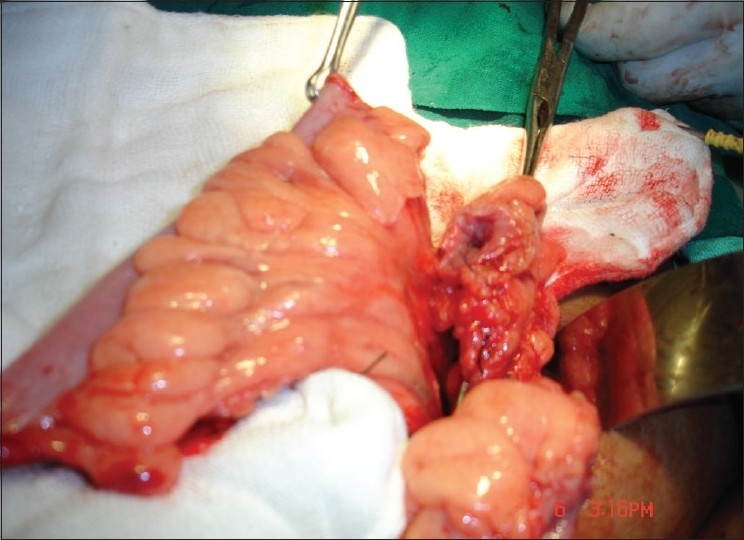
Peroperative photograph showing sigmoid segment on its vascular pedicle

**Figure 2:**
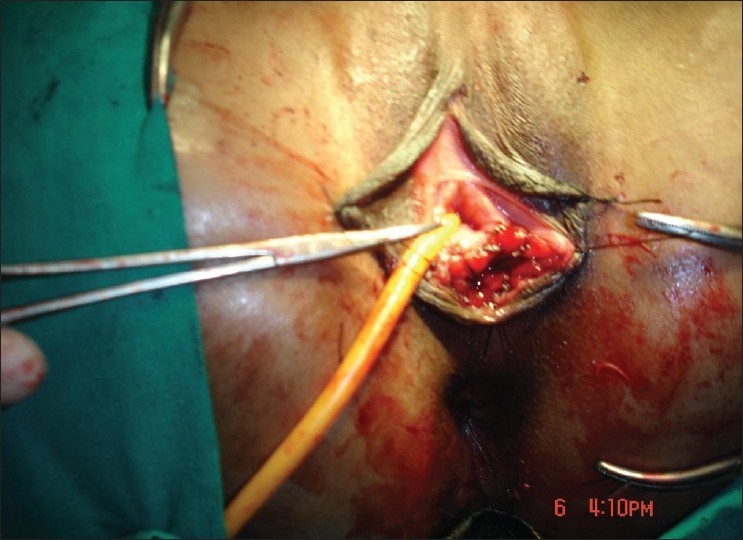
Immediate postoperative picture showing newly created vagina

The approach was abdominoperineal in all the patients. Pfannenstiel or lower midline longitudinal incision was made to open the abdomen. After opening the abdomen, about 10–15cm long segment of sigmoid colon was mobilized on its vascular pedicle [Figure 1]. Vascular pedicle plane of the sigmoid graft was taken in between the left colic and the superior rectal artery. Sigmoid graft had at least 2 sigmoidal arteries for better survival. The continuity of the colon was restored by a hand-sewn double-layer end-to-end anastomosis using 3-0 absorbable sutures. Depending on the individual patient’s vascular anatomy, either an isoperistaltic or antiperistaltic orientation was used. The proximal end of the sigmoid segment was closed in 2 layers. Lower end of the graft was brought down to the perineum, after creating a rectovesical tunnel between urethra, bladder anteriorly, and rectum posteriorly. The edges of the vaginal pit were sutured to the distal end of the sigmoid segment and the lumen packed with gauze pieces for 1–2 days postoperatively. A Foley’s catheter was kept in the bladder for 4 days. At discharge from the hospital, the patients were instructed to irrigate the neovagina daily for 8 weeks and weekly thereafter [Figure 2].

## RESULTS

The total duration of this study was 7 years. The total number of patients included in this study were 8. The age range was 12–17 years (mean 14.4 years). All the patients presented with amenorrhea. The mean follow-up duration was 4.2 years. One patient was lost to follow-up. None of the 8 patients had any significant peroperative complication. Two patients had prolonged postoperative ileus, managed conservatively. One patient had minor abdominal wound infection, which responded well to local wound care and systemic antibiotics. One patient had mucosal prolapse of the neovagina that required trimming.

Neovagina was cosmetically acceptable to the parents of the 7 patients. One patient had unacceptable appearance of vulva due to badly scarred perineum, which was not up to the mark cosmetically. None of the patients had vaginal stenosis during follow-up visits. Also, none of them had excessive mucus discharge. Out of these 7 patients who are in regular follow-up, 2 are sexually active and satisfied. Although postsurgical results are acceptable to the parents and patients cosmetically, the long-term sexual and psychologic outcomes are yet to be assessed.

## DISCUSSION

Vaginal atresia due to Müllerian aplasia is found in patients with MRKH syndrome.[7] Surgical treatment of this patient population constitutes a significant technical challenge, the outcome of which affects both the physical and psychosocial health of the patient. The ideal reconstructive procedure should provide a vagina that has an appropriate length and that requires minimal, if any dilatation. It should not scar, stenose, or contract and should provide a satisfactory cosmetic result, leaving the external genitalia intact.[8]

The traditional treatment for this condition is the split-thickness skin graft.[1] This procedure has high rates of graft failure and stenosis. Frequent dilatations and need to wear a vaginal mold at night are other disadvantages. Incidence of dyspareunia directly correlates with the length of the neovagina, with an incidence of 100% if the vaginal length is less than 6 cm.[9] It is extremely difficult to have good length of vagina after few years of surgery (traditional) and even after having a good length, vagina is dry and painful on coitus.[9]

Colon, cecum, or ileum may be used for bowel vaginoplasty but the sigmoid colon is preferred over the others because it satisfies the following criteria according to Rajimwale *et al*.: (1) it is self-lubricating; (2) mucus production is less of a problem than with the use of the small bowel; (3) it can grow with the child when used to create a neovagina before puberty; (4) there is a minimal risk of stenosis; (5) it is close to the perineum; (6) it has an easily mobilized vascular pedicle; and (7) it does not require moulds or stenting.[10] Sigmoid vaginoplasty provides an esthetically pleasing neovagina with a good length, natural lubrication, and obviating the need for stenting and/or dilatation.[10] Laparoscopic approaches for sigmoid vaginal reconstruction were tried.[11,12] Karateke *et al* advised that the preparation of the sigmoid flap should be individualized according to the length of the sigmoid flap and mesosigmoid together with the distribution of the sigmoid arteries and their relation with the left colic and superior rectal arteries.[3]

None of the patients in our series complained of local irritation, dryness, or dyspareunia. Excessive mucus discharge was reported in the initial months after surgery but subsided after 3–4 months, similar to that reported by Hanna.[13] Mucosal prolapse is one of the late postoperative complications that occurred in one of our case, which was successfully treated by excision. Parsons *et al*. reported 4 (14%) cases out of 28 with mucosal prolapse successfully treated by local excision and fulguration.[14] Fixation of the sigmoid neovagina to the sacral promontory and/or the pelvic floor may lessen the occurrence of this complication. It was done in all of our patients.

Psychosexual profiles, as determined by personal interview of these patients, indicated that most of the patients achieved acceptable outcomes with respect to the physical appearance of the vagina. Out of 7 patients who are in regular follow-up, 2 are sexually active and satisfied.

None of the patients in our series had any major postoperative or chronic complications. However, certain potential complications merit discussion. Stenosis of the mucocutaneous junction has been reported.[15] However, we did not encounter any such stenosis in our patients. Ulcerative colitis has been reported in the neovagina.[16] Similarly, patients with hereditary polyposis syndromes, such as familial polyposis, Gardner syndrome, and nonpolypous colon cancer have the potential to develop polyps or neoplasia in the diverted sigmoid colon. Another potential complication of the sigmoid neovagina is diversion colitis, a rare disorder of unknown etiology occurring after isolation of an intestinal segment from the fecal stream.[17] None of the patients in our series had these complications; this could have been because of the low incidence of ulcerative colitis in the population in the Indian subcontinent.

However, continuous follow-up of these patients with regard to these potential complications is necessary.

## CONCLUSION

Sigmoid vaginoplasty is a safe and acceptable procedure to treat the patients of vaginal agenesis with acceptable cosmetic results and complication rate. We suggest sigmoid colon vaginoplasty as a better treatment modality because of its large lumen, thick wall resistant to trauma, adequate secretion allowing lubrication, not necessitating prolonged dilatation, and short recovery time.

## References

[1] (2006). ACOG Committee Opinion No. 355. Vaginal agenesis, diagnosis, management, and routine care. Obstet Gynecol.

[2] Grosfeld JL, Coran AG, Grosfeld JL, O–Neill AJ, Fonkalsrud EW, Coran AG (2006). Abnormalities of the female genital tract. Textbook of Pediatric surgery.

[3] Karateke A, Gurbuz A, Haliloglu B, Kabaca C, Koksal Nnone (2006). Intestinal vaginoplasty: Is it optimal treatment of vaginal agenesis? A pilot study. Surgical method of sigmoid colon vaginoplasty in vaginal agenesis. Int Urogynecol J Pelvic Floor Dysfunct.

[4] Ashworth MF, Morton KE, Dewhurst J, Lilford RJ, Bates RG (1986). Vaginoplasty using amnion. Obstet Gynecol.

[5] Jackson ND, Rosenblatt PL (1984). Use of interceed absorbable barrier for vaginoplasty. Obstet Gynecol.

[6] Hendren WH, Atala A (1994). Use of bowel for vaginal reconstruction. J Urol.

[7] Kapoor R, Sharma DK, Singh KJ, Suri A, Singh P, Chaudhary H (2006). Sigmoid vaginoplasty: Long-term results. Urology.

[8] Hensle TW, Chang DT (1999). Vaginal reconstruction. Urol Clin North Am.

[9] de Souza AZ, Maluf M, Perin PM, Maluf Filho F, Perin LF (1987). Surgical treatment of congenital uterovaginal agenesis: Mayer–Rokitansky–Kuster–Hauser syndrome. Int Surg.

[10] Rajimwale A, Furness PD, Brant WO, Koyle MA (2004). Vaginal construction using sigmoid colon in children and young adults. BJU Int.

[11] Darai E, Toullalan O, Besse O, Potiron L, Delga P (2003). Anatomic and functional results of laparoscopic-perineal neovagina construction by sigmoid colpoplasty in women with Rokitansky’s syndrome. Hum Reprod.

[12] Urbanowicz W, Starzyk J, Sulislawski J (2004). Laparoscopic vaginal reconstruction using a sigmoid colon segment: A preliminary report. J Urol.

[13] Hanna MK (1987). Vaginal construction. Urology.

[14] Parsons JK, Gearhart SL, Gearhart JP (2002). Vaginal reconstruction utilizing sigmoid colon: Complications and long-term results. J Pediatr Surg.

[15] Tillem SM, Stock JA, Hanna MK (1998). Vaginal construction in children. J Urol.

[16] Froese DP, Haggitt RC, Friend WG (1991). Ulcerative colitis in the autotransplanted neovagina. Gastroenterology.

[17] Toolenaar TA, Freundt I, Huikeshoven FJ, Drogendijk AC, Jeekel H, Chadha AS (1993). The occurrence of diversion colitis in patients with a sigmoid neovagina. Hum Pathol.

